# On Medical Provision for Railroads, as a Humanitarian Measure, as Well as a Source of Economy to the Companies

**Published:** 1862-11

**Authors:** 


ON MEDICAL PROVISION FOR RAILROADS, AS A HUMANITARIAN MEASURE, AS WELL AS A SOURCE OF ECONOMY TO THE COMPANIES. By EDMUND S. F. ARNOLD, M.D., Fellow of the New York Academy of Medicine, &c., &c. New York: BAILLIERE BROTHERS, 440 Broadway. 1862.

This is a pamphlet of 47 pages, embracing two papers; one read before the New York State Medical Society, Feb. 5th, '62, and the other before the Surgical Section of the New York Academy of Medicine, Oct. 28, 1862. The subject discussed in these papers, namely, the establishment of better provisions for the care of persons injured on railroads, is certainly worthy of careful consideration. The author has evidently bestowed upon it much attention, and his papers should be extensively circulated and read.

The outline of the plan advocated by Dr. ARNOLD, is contained in the three following resolutions, appended to the paper read to the Surgical Section of the Academy of Medicine, and adopted by that Section, viz.:—

1st. That in the opinion of this body the loss of many lives would be avoided, and a great diminution of suffering brought about by the adoption of a system of local medical provision on our railroads to meet the cases of casualty occurring thereon.

2d. That such a medical provision could readily be made by fitting up a small room at main stations, and at distances not more than ten miles apart, where practicable, for temporary hospital purposes, and by providing the same with suitable apparatus, and appointing a competent surgeon in the neighborhood to attend on call as occasion may arise.

3d. That, when important stations are too far apart to admit of such an arrangement, suitable apparatus should be carried in the cars themselves, and stations be established where the most competent practitioners of surgery are to be found.

